# Factors affecting institutional delivery in rural Chitwan district of Nepal: a community-based cross-sectional study

**DOI:** 10.1186/s12884-015-0454-y

**Published:** 2015-02-13

**Authors:** Rajani Shah, Eva A Rehfuess, Mahesh K Maskey, Rainald Fischer, Prem B Bhandari, Maria Delius

**Affiliations:** Center for International Health, Ludwig-Maximilians-University, Munich, Germany; Institute for Medical Informatics, Biometry and Epidemiology, Ludwig-Maximilians- University, Munich, Germany; Nepal Public Health Foundation, Kathmandu, Nepal; Pneumologische Praxis München-Pasing, Munich, Germany; Population Studies Center, University of Michigan, Ann Arbor, USA; Department of Gynaecology and Obstetrics, Ludwig-Maximilians-University, Munich, Germany

**Keywords:** Institutional delivery, Birthing centre, Women’s empowerment, Maternal health, Nepal, South Asia

## Abstract

**Background:**

Health facility delivery is considered a critical strategy to improve maternal health. The Government of Nepal is promoting institutional delivery through different incentive programmes and the establishment of birthing centres. This study aimed to identify the socio-demographic, socio-cultural, and health service-related factors influencing institutional delivery uptake in rural areas of Chitwan district, where high rates of institutional deliveries co-exist with a significant proportion of home deliveries.

**Methods:**

This community-based cross-sectional study was conducted in six rural Village Development Committees of Chitwan district, which are characterised by relatively low institutional delivery rates and the availability of birthing centres. The study area represents both hilly and plain areas of Chitwan. A total of 673 mothers who had given birth during a one-year-period were interviewed using a structured questionnaire. Univariate and multivariable logistic regression analysis using stepwise backward elimination was performed to identify key factors affecting institutional delivery.

**Results:**

Adjusting for all other factors in the final model, advantaged caste/ethnicity [aOR: 1.98; 95% CI: 1.15-3.42], support for institutional delivery by the husband [aOR: 19.85; 95% CI: 8.53-46.21], the decision on place of delivery taken jointly by women and family members [aOR: 5.43; 95% CI: 2.91-10.16] or by family members alone [aOR: 4.61; 95% CI: 2.56-8.28], birth preparations [aOR: 1.75; 95% CI: 1.04-2.92], complications during the most recent pregnancy/delivery [aOR: 2.88; 95% CI: 1.67-4.98], a perception that skilled health workers are always available [aOR: 2.70; 95% CI: 1.20-6.07] and a birthing facility located within one hour’s travelling distance [aOR: 2.15; 95% CI: 1.26-3.69] significantly increased the likelihood of institutional delivery. On the other hand, not knowing about the adequacy of physical facilities significantly decreased the likelihood of institutional delivery [aOR: 0.14; 95% CI: 0.05-0.41].

**Conclusion:**

With multiple incentives present, the decision to deliver in a health facility is affected by a complex interplay of socio-demographic, socio-cultural, and health service-related factors. Family decision-making roles and a husband’s support for institutional delivery exert a particularly strong influence on the place of delivery, and this should be emphasized in the health policy as well as development and implementation of maternal health programmes in Nepal.

## Background

Improving maternal health is one of the United Nation’s Millennium Development Goals (MDG 5) with a target of reducing the maternal mortality ratio (MMR) by three quarters by 2015 from its 1990 level [1]. Globally, 289,000 maternal deaths occurred in 2013. Almost all the maternal deaths (99 percent) occurred in developing countries [2]. Continuous care during pregnancy, delivery and the postpartum period is essential for maternal and newborn health. The most risky period for mother and child is during child birth and the first few days postpartum [3]. Between 1996 and 2006, the MMR in Nepal dropped from 539 to 281 per 100,000 live births [4] and to 190 per 100,000 live births in 2013 [2]. However, the MMR of Nepal is still highest in the South Asian countries except Afghanistan [2].

In a country like Nepal, the chances of a safe delivery are greater when the birth takes place in a health facility than at home, and increasing institutional delivery is important to reduce deaths due to pregnancy complications [5,6]. Although there has been a significant rise in institutional delivery in the past 10 years, with an increase from 9 percent in 2001 to 35 percent in 2011, nearly two thirds of births in Nepal (65 percent) continue to take place at home [7]. Thus, encouraging institutional delivery and 24-hour emergency obstetric care services at selected public health facilities in every district is one of the major strategies Nepal has adopted to reduce the risk of dying during childbirth [8]. A safe birth includes providing supportive company, ensuring clean delivery practices, as well as early detection and management of maternal and neonatal complications. Life-saving packages consisting of medications and surgical material should be available at every birth and access to operative vaginal delivery, to caesarean section, and to blood transfusion should be within a reachable distance [5,6]. Thus, the Nepali government has added new birthing centres within health posts/sub-health posts to increase the number of institutional deliveries [8]. These centers provide a 24-hour service to manage uncomplicated deliveries and to refer complicated cases to hospitals [8]. In addition, under the Safer Mother Programme, which came into effect in 2009, handling of all deliveries with or without complications is provided free of charge at all those governmental, private as well as NGO-run health facilities and teaching hospitals that have received permission from the Government of Nepal [9]. Moreover, a cash incentive provided to mothers after having given birth at a health facility forms part of a safe delivery incentive programme since 2005 [8]. Additionally, in programmes such as the Community Based Newborn Care Program (CB-NCP), female community health volunteers (FCHVs) identify and counsel pregnant women and encourage them to deliver in a health institution [10].

Chitwan district, the study setting, is located in the southern plain (terai) of Nepal (with some hilly areas) and is characterized by a relatively better health and road infrastructure than other districts. As a result, the percentage of institutional deliveries is much higher (83 percent) [11] compared to the nation as a whole (35 percent) [7]. Nevertheless a significant proportion of deliveries take place at home, especially in rural areas. Thus, this setting is particularly suitable for examining the factors that favour or limit institutional delivery, and to derive lessons learnt that may be applicable to other districts across the country. This study aims to identify socio-demographic, socio-cultural, and health service-related factors influencing institutional delivery in rural areas of Chitwan district.

## Methods

### Study setting

Geographically, Nepal is characterized by three ecological regions - mountain, hill and plain. The country is divided into 75 districts, and each district is structured in Village Development Committees (VDCs) and municipalities that are classified as rural and urban respectively [12]. Each VDC and municipality is further divided into smaller administrative units called wards. There are nine wards in each VDC, while the number varies for municipalities.

Chitwan district is located 148 kilometers far from the capital Kathmandu. The district comprises a total of 36 VDCs, 9 located in the hills and 27 located in the plain [11], and 2 municipalities with a total population of 579,984 [13]. According to the National Population and Housing Census 2011, the distribution of different caste/ethnicities in the district is as follows: upper caste (Brahman and Chhetri) 41 percent; advantaged Janajati 12 percent; Dalit 8 percent; and disadvantaged Janajati 39 percent [14]. About two thirds of the population aged five years and above are literate, with three quarters of males compared to 57 percent of females [15]. One third of males do not live at home, with most of these working abroad [15]. A composite index based on poverty and deprivation, socio-economic and infrastructural development, and women’s empowerment shows Chitwan to be the second most developed district in Nepal [16].

There are several hospitals in Chitwan: one government sub-regional hospital, two teaching hospitals and 36 private hospitals. Most of them are located in the district headquarter Bharatpur and with birthing facilities. In rural areas, health services are provided through four primary health care centers (PHCCs), 24 health posts (HPs) and 12 sub-health posts (SHPs) [11].

### Study design, study participants and sample size

This community-based cross-sectional study included all women who had given birth during the one-year period between April 21, 2012 and April 20, 2013 (i.e. one week prior to the start of data collection, which allowed FCHVs one week to record the birth).

**Figure 1:**
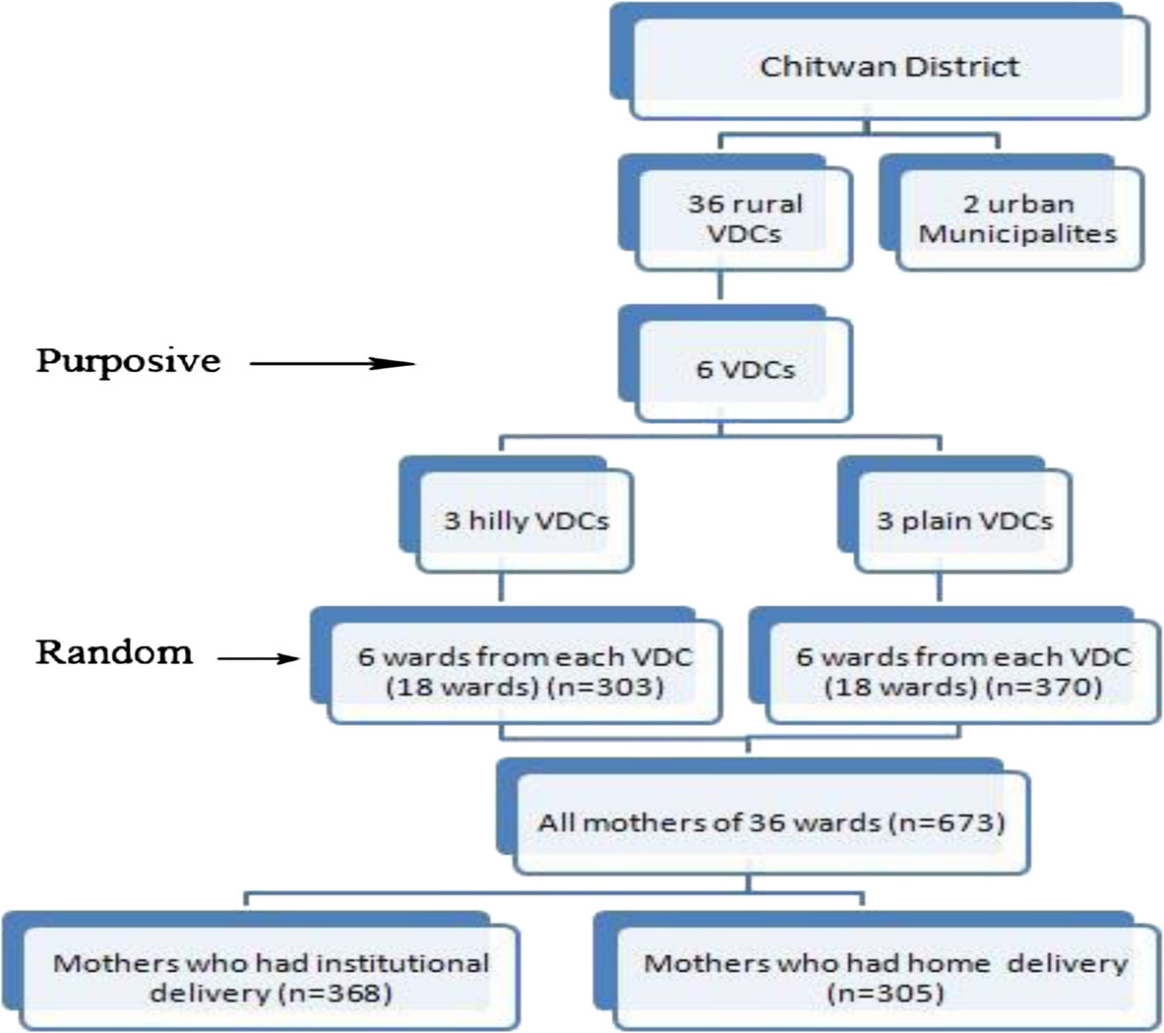
**Sampling procedure and sample size.**

First, six rural VDCs - 3 hilly (Kaule and Chandivanjyang with birthing centres and Kabilas without a birthing centre) and 3 plain (Piple and Ayodhyapuri with birthing centres and Padampur without a birthing centre) VDCs - were selected on the basis of a lower-than-district average percentage of institutional delivery and the availability of birthing facilities. In the six selected VDCs, the total female population of reproductive age (15–49 years) was 17,690 and the expected pregnancies were 1891 [11]. Secondly, in each VDC, six out of nine wards were selected randomly, with two thirds of wards assumed to be representative of the VDC as a whole and generating a sufficiently large number of deliveries. Finally, at ward level, all mothers having given birth during the specified one-year-period and residing in the ward were interviewed. The sampling process is shown in Figure 1.

The sample size for the study was calculated with the average 46 percent prevalence of institutional delivery of the six selected VDCs as reported by the CB-NCP [unpublished observation], 4 percent margin of error, and assuming a non-response rate of 10 percent. This resulted in a final sample size of 663, allowing for sufficient power even in view of the many questions asked through the survey [17]. Altogether 673 mothers were included, among whom 368 had delivered at a health facility and 305 had delivered at home (Figure 1).

### Data collection

Data collection took place between April 26, 2013 and June 30, 2013. Chitwan is one of the districts where the CB-NCP is implemented. FCHVs keep a record of all pregnant women and mothers who have given birth. The FCHVs of the selected wards were visited at their homes and a list of mothers who had given birth between April 21, 2012 and April 20, 2013 was developed from their records. In addition, other eligible mothers of the study area not reached by the FCHVs, primarily due to geographical or distance reasons, were also included in our study. These were identified by asking the FCHVs themselves, as well as other local residents.

A structured questionnaire was prepared in the local Nepali language on the basis of a review of the relevant literatures and the preliminary analyses of separately conducted focus group discussions and in depth interviews (manuscript in preparation). The questionnaire included socio-economic, demographic and cultural variables related to the role of family and neighbours, variables related to perceived need for health care, and health service-related variables. It was pre-tested among 35 women residing in three other rural VDCs of Chitwan district that are similar to those of the study area. The feedbacks from the pre-test were incorporated and questionnaire was then finalized. Modifications included a re-ordering of questions as well as changes in words and additions/deletions of some answer categories.

Data collection took place through face-to-face interviews with the participating mothers by visiting them at their homes. Eight experienced enumerators, Bachelor students of public health and Master students of sociology, were selected to collect the data. For two VDCs, the enumerators were recruited locally, while for the other VDCs most of the enumerators were not local residents but familiar with the area. The first author provided them with two days’ training, and they were also involved in the pretesting of the questionnaire.

### Variables

Table 1 shows the construction of dependent and independent variables in major groups (i.e. socio-economic and socio-demographic variables, socio-cultural factors including the role of family and neighbours and perceived need-related variables, perceived health service-related variables and distance to birthing facility); where needed, additional description of variables is provided. The dependent/outcome variable, i.e. place of delivery, assesses whether the most recent child birth occurred at a health institution (coded 1) or at home (coded 0).

**Table 1:** **Definition of variables**

**Variables**		**Measurements**
**Outcome variable**		
1	Place of delivery	Institutional delivery (coded 1), Home delivery (coded 0)
**Socio-economic and demographic variables**		
2	Place of residence	Plain, Hill*
3	Caste/ethnicity	Disadvantaged*, Advantaged
4	Wealth index	Poorer*, Better
5	Maternal educational status	No education*, Primary education, Secondary or higher education
6	Maternal age	15-19 years*, 20–29 years, 30 years and above
7	Birth order	1st, 2nd-3rd, 4th or more*
**Variables related to role of family and neighbours**		
8	Final decision-maker	Woman alone*, Woman and family members jointly, Family members alone/FCHV
9	Support of husband	Encourages home delivery*, Encourages institutional delivery, No response
10	Support of neighbours	Encourage home delivery*, Encourage institutional delivery, No response
**Perceived need-related variables**		
11	Birth preparation	Preparation (1 or more preparations achieved), No preparation*
12	Number of antenatal care (ANC) check-ups	4 or more, <4, No ANC*
13	Experience of complications during the most recent pregnancy or childbirth	Yes, No*
**Perceived health service-related variables**		
14	Availability of skilled health worker	Sometimes*, Always, Don’t know
15	Health worker care and respect	Sometimes*, Always, Don’t know
16	Availability of drugs and equipment	Sometimes*, Always, Don’t know
17	Adequacy of physical facilities	Insufficient*, Sufficient, Don’t know
**Distance to birthing facility**		
18	Distance to birthing facility	<1 hour, 1 hour or more*

*Indicates the reference category.

#### Caste/Ethnicity

The caste-ethnicity variable reflects the important socio-cultural structure of Nepali society [18-20]. The hierarchical caste system is fundamental to the Hindu religion. Upper caste Hindus (e.g., Brahman and Chhetri) are at the top of the social hierarchy and are presumed to be socio-culturally, economically and politically advantaged compared to other caste/ethnic groups [20,21]. Historically, Dalits (e.g., Kami, Sunar, Damai, Sarki) are untouchables and positioned at the bottom of the social hierarchy. Janajatis (e.g. Gurung, Tamang, Magar, Newar) may also have hierarchy within their system, but the social hierarchy is not as distinct as in other caste groups. The upper caste Hindu and Newar people are among the historically privileged groups and are considered the elites [20,22]. It is believed that these groups, particularly the upper caste Hindu, have the best access to various economic and non-economic opportunities [23].

The Ministry of Health and Population of Nepal has classified the different caste/ethnicities into six categories: upper caste group (Brahman/Chhetri), relatively advantaged Janjatis, disadvantaged Janjatis, disadvantaged non-Dalit terai caste group, Dalits, and religious minorities [24]. In the present study, the caste/ethnicity variable was dichotomized into an advantaged group (i.e. upper castes (Brahman/Chhetri) and advantaged Janajatis) and a disadvantaged group (i.e. disadvantaged Janajatis, Dalits, and religious minorities) [25].

#### Wealth index

A wealth index was created using a principal component analysis of a number of household assets [26], i.e. possession of a radio, television, mobile, other kind of telephone, and watch; dwelling characteristics - material of roof and wall of house, availability of a toilet, type of toilet and access to drinking water and electricity. The factor score from the first component of the principal component analysis was used to classify respondents with poorer and better wealth index.

#### Education

Education is measured in three categories - no education, primary and secondary. Women who had completed any of the grades from 1 to 5 were classified as having primary education. Women who had completed six grades or more were classified as having secondary or higher education.

#### Birth order

The order of the recent birth was categorized as ‘first’ , ‘second or third’ , ‘fourth or more’.

#### Final decision-maker

The final decision regarding place of delivery was categorized as woman alone, woman and family members jointly and family members and FCHVs. The FCHV category was merged with other family members as its frequency was very low, i.e., four out of 673.

#### Support of husband

This variable assessed verbal or physical support by the husband towards institutional delivery, such as carrying the woman to the health facility, arranging for transportation, or saving money.

#### Support of neighbours

This variable assessed verbal or physical support by neighbours towards institutional delivery, such as help in carrying the woman to the health facility.

#### Birth preparation

Women were considered prepared for birth when at least one of five components of birth preparation, i.e. saving money, arranging transportation, identifying health institution and contacting a health worker, identifying a person who can donate blood in case of need, and owning a clean delivery kit [7], had been achieved.

#### Number of antenatal care (ANC) check-ups

The number of antenatal care check-ups a woman had during her recent pregnancy was counted. The response is grouped as – no visits, less than 4 visits, and 4 or more visits.

#### Experience of complications

Perceived experience of complications during the recent pregnancy or childbirth measured as a dichotomy (yes = 1; no = 0).

#### Availability of skilled health worker

This variable measures how women perceived the availability of skilled health workers at the nearest birthing facility (a birthing centre or hospital).

#### Care and respect from health workers

This variable measures how women perceived the care and respect of the health workers at the nearest birthing facility.

#### Availability of drugs and equipment

This variable measures the perceived availability of the necessary drugs and equipment at the nearest birthing facility.

#### Adequacy of physical facilities

This variable measures the perceived adequacy of the physical facilities/infrastructure at the nearest birthing facility including a waiting room, a bed room, a room for delivery, light and water.

#### Distance to birthing facility

The distance to the birthing facility is measured as the time required to reach the nearest birthing facility with the available means of transportation, whether by vehicle, bamboo basket, and hammock or by walking.

### Data processing and analysis

Data entry was undertaken in Epi data version 3.1 [27]. Analyses were conducted in SPSS version 16 [28]. The outcome of interest was place of delivery, coded as a binary variable. We initially conducted univariate logistic regression analyses to obtain unadjusted odds ratios for the effect of a broad range of variables on institutional delivery. We then used multivariable logistic regression analysis and stepwise backward elimination, where all independent variables are entered in the initial model and at each step the variable with the least significance (the highest p-value greater than 0.05) is eliminated. The final model, which is the reduced model, included all the variables showing significant associations with place of delivery [29].

To diagnose potential multi-collinearity problems among the independent variables, a collinearity diagnostic test available in SPSS was used. A tolerance statistic above 0.2 and a variance inflation factor (VIF) value above 5.0 were considered to indicate multi-collinearity [30]. However, all the VIF values were well below 3.0.

### Ethical considerations

The Nepal Health Research Council granted ethical clearance to carry out this study. We obtained a written permission from the District Public Health Office in Chitwan to implement this study in Chitwan. An informed consent form was prepared on the basis of templates made available by the World Health Organization (http://www.who.int/rpc/research_ethics/informed_consent/en/). Prior to the beginning of the interviews, we obtained verbal consent from the respondents as most of them were illiterate. Verbal informed consent was obtained from the women or from their guardian/parent/next of kin if the women’s age was below the legal age, for the publication of this report and any accompanying images.

## Results

### Description of study participants

Among the 673 women participating in the study, 55% (n = 368) had delivered at a health facility and the remaining 45% (n = 305) had delivered at home (Table 2). This institutional delivery rate is significantly below the average for Chitwan district (83%), which is a direct consequence of the confinement of the study area to rural areas.

**Table 2:** **Distribution of independent variables across home vs. health facility delivery**

**Variable**	**Place of delivery**		
	**Home**	**Health facility**	**Total**
	**(n = 305) (45%)**	**(n = 368) (55%)**	**(N = 673) (100%)**
**Socio-economic and Demographic Variables**			
**Place of residence**			
Plain	113 (31)	257 (70)	370
Hill	192 (63)	111 (37)	303
**Caste/ethnicity**			
Disadvantaged caste	260 (54)	219 (46)	479
Advantaged caste	45 (23)	149 (77)	194
**Wealth index**			
Poorer	221 (66)	115 (34)	336
Better	84 (25)	253 (75)	337
**Maternal educational status**			
No education	133 (70)	57 (30)	190
Primary education	104 (51)	99 (49)	203
Secondary or higher education	68 (24)	212 (76)	280
**Maternal age**			
15-19 years	59 (37)	101 (63)	160
20-29 years	206 (46)	239 (54)	445
30 years and above	40 (59)	28 (41)	68
**Birth order**			
1^st^	81 (28)	206 (72)	287
2nd or 3^rd^	139 (50)	139 (50)	278
4th or more	85 (79)	23 (21)	108
**Variables Related to Role of Family and Neighbours**			
**Final decision-maker**			
Woman alone	203 (72)	81 (29)	284
Woman and family members jointly	65 (32)	137 (68)	202
Family members alone/FCHV	37 (20)	150 (80)	187
**Support of husband**			
Encourages home delivery	125 (93)	9 (7)	134
Encourages institutional delivery	106 (24)	342 (76)	448
No response	74 (81)	17 (19)	91
**Support of neighbours**			
Encourage home delivery	125(86)	21 (14)	146
Encourage institutional delivery	124 (28)	319 (72)	443
No response	56 (67)	28 (33)	84
**Perceived Need-related Variables**			
**Birth preparation**			
No preparation	204 (66)	103 (34)	307
Preparation	101 (28)	265 (72)	366
**Number of ANC check-ups**			
1 to 3	179 (59)	125 (41)	304
4 or more	66 (22)	234 (78)	300
No ANC	60 (87)	9 (13)	69
**Experience of complications**			
No	252 (52)	237 (49)	489
Yes	53 (29)	131 (71)	184
**Perceived Health Service-Related Variables**			
**Availability of skilled health worker**			
Sometimes	56 (71)	23 (29)	79
Always	170 (34)	338 (67)	508
Don’t know	79 (92)	7 (8)	86
**Health worker care and respect**			
Sometimes	42 (65)	23 (35)	65
Always	184 (35)	337 (65)	521
Don’t know	79 (91)	8 (9)	87
**Availability of drugs and equipment**			
Sometimes	76 (38)	125 (62)	201
Always	107 (32)	227 (68)	334
Don’t know	122 (88)	16 (12)	138
**Adequacy of physical facilities**			
Insufficient	76 (38)	125 (62)	201
Sufficient	107 (32)	227 (68)	334
Don’t know	122 (88)	16 (12)	138
**Distance to Birthing Facility**			
<1 hour	94 (24)	302 (76)	396
1 hour or more	211 (76)	66 (24)	277

Table 2 also shows the distribution of independent variables. Among the socio-economic and socio-demographic factors, a higher proportion of younger women living in VDCs in plain areas, of advantaged caste/ethnicities, with better wealth index and having secondary or higher education gave birth at a health facility. Similarly, as expected, the percentage of institutional delivery decreased with increasing birth order. With respect to the role of family and neighbours, women who were encouraged to deliver at a health institution by their husbands were more likely to do so than women who were not encouraged by their husbands. However, unexpectedly, those women who decided on place of delivery by themselves were more likely to deliver at home. With respect to perceived need, a higher proportion of women who had prepared for birth, had had antenatal check-ups or experienced complications during the current pregnancy/delivery gave birth at a health facility. Similarly, a higher proportion of those women who reported that health workers were always available, always care and respect patients, and that drugs and equipment were always available, and physical facilities were adequate gave birth at a health facility. More women living within one hour’s distance gave birth at a health facility.

### Multivariable analysis

Table 3 shows the results of univariate and multivariable logistic regression. In univariate models, all variables were statistically significantly associated with the outcome. However, adjusted odds ratios from the multivariable analysis are reported only for those variables that are statistically significantly associated with the place of delivery and are included in the final model.

**Table 3:** **Results of logistic regression analysis**

**Variables**	**Unadjusted OR (95% CI)**	**Adjusted OR (95% CI)**
**Socio-economic and Demographic Variables**		
Place of residence		**NS**
Hill	1.00	-
Plain	**3.93 (2.85-5.43)**	-
	(p < 0.001)	
**Ethnicity**		**-**
Disadvantaged caste	1.00	1.00
Advantaged caste	**3.93 (2.69-5.74)**	**1.98 (1.15-3.42)**
	(p < 0.001)	(p = 0.014)
**Wealth index**		NS
Poorer	1.00	**-**
Better	**5.79 (4.14-8.09)**	**-**
	(p < 0.001)	
**Maternal educational status**		NS
No education	1.00	**-**
Primary education	**2.22 (1.47-3.36)**	**-**
	(p < 0.001)	
Secondary or higher education	**7.28 (4.81-11.00)**	**-**
	(p < 0.001)	
**Maternal age**		NS
15-19 years	1.00	**-**
20-29 years	**0.68 (0.47-0.98)**	**-**
	(p = 0.04)	
30 years and above	**0.41 (0.23-0.73)**	**-**
	(p = 0.003)	
**Birth order**		NS
1^st^	**9.40 (5.55-15.93)**	**-**
	(p < 0.001)	
2nd to 3^rd^	**3.70 (2.20-6.20)**	**-**
	(p < 0.001)	
4th or more	1.00	**-**
**Variables Related to Role of Family and Neighbours**		
**Final decision-maker**		
Woman herself	1.00	1.00
Woman and family members jointly	**5.28 (3.57-7.82)**	**5.43 (2.91-10.16)**
	(p < 0.001)	(p < 0.001)
Other family members/FCHV	**10.16 (6.53-15.82)**	**4.61 (2.56-8.28)**
	(p < 0.001)	(p < 0.001)
**Support of husband**		
Encourages home delivery	1.00	1.00
Encourages institutional delivery	**44.81 (22.02-91.21)**	**19.85 (8.53-46.21)**
	(p < 0.001)	(p < 0.001)
No response	**3.19 (1.35-7.52)**	2.85 (0.99-8.22)
	(p = 0.008)	(p = 0.52)
**Support of neighbours**		NS
Encourage home delivery	1.00	**-**
Encourage institutional delivery	**15.31 (9.23-25.42)**	**-**
	(p < 0.001)	
No response	**2.98 (1.56-5.69)**	**-**
	(p = 0.001)	
**Perceived Need-related Variables**		
**Birth preparation**		
No preparation	1.00	1.00
Preparation	**5.20 (3.74-7.23)**	**1.75 (1.04-2.92)**
	(p < 0.001)	(p = 0.034)
**Number of ANC check-ups**		NS
1 to 3	**4.66 (2.23-9.73)**	**-**
	(p < 0.001)	
4 or more	**23.64 (11.14-50.14)** (p < 0.001)	**-**
No ANC	1.00	**-**
**Experience of complications**		
No	1.00	1.00
Yes	**2.63 (1.82-3.79)**	**2.88 (1.67-4.98)**
	(p < 0.001)	(p < 0.001)
**Perceived Health Service-related Variables**		
**Availability of skilled health worker**		
Sometimes	1.00	1.00
Always	**4.84 (2.88-8.14)**	**2.70 (1.20-6.07)**
	(p < 0.001)	(p = 0.016)
Don’t know	**0.22 (0.09-0.54)**	2.46 (0.54-11.22)
	(p = 0.001)	(p = 0.247)
**Health worker care and respect**		NS
Sometimes	1.00	-
Always	**3.35 (1.95-5.74)**	-
	(p < 0.001)	
Don’t know	**0.19 (0.08-0.45)**	-
	(p < 0.001)	
**Availability of drugs and equipment**		NS
Sometimes	1.00	-
Always	1.41 (0.98-2.03)	-
	(p = 0.07)	
Don’t know	**0.08 (0.04-0.18)**	-
	(p < 0.001)	
**Adequacy of physical facilities**		
Insufficient	1.00	1.00
Sufficient	1.29 (0.89-1.86)	1.04 (0.59-1.84)
	(p < 0.173)	(p = 0.892)
Don’t know	**0.08 (0.04-0.14)**	**0.14 (0.05-0.41)**
	(p < 0.001)	(p < 0.001)
**Distance to Birthing Facility**		
<1 hour	**10.27 (7.16-14.73)**	**2.15 (1.26-3.69)**
	(p < 0.001)	(p = 0.005)
1 hour or more	1.00	1.00
**Nagelkerke’s R-square**		**0.673**
**Hosmer and lemeshow statistics**		**0.439**

NS, variable not selected in multivariable model.

Bold font indicates statistical significance with p < 0.05.

Of the various socio-economic and socio-demographic variables in the analysis, only the effect of caste/ethnicity remained statistically significant in the multivariable analysis. Adjusting for the effect of all other variables in the final model, mothers of advantaged caste/ethnicity were about two times (adjusted OR 1.98; 95% CI 1.15-3.42) more likely than mothers of disadvantaged caste/ethnicity to deliver at a health facility.

Interestingly, mothers were more likely to deliver at a health facility if the decision was taken jointly by the mother and family members [aOR: 5.43; 95% CI: 2.91-10.16], or by other family members and/or FCHVs [aOR: 4.61; 95% CI: 2.56-8.28] compared to the decision taken by the women alone. Encouragement from the husband was very important for the decision. Mothers who were supported by their husbands to deliver at a health facility being about 20 times [aOR: 19.89; 95% CI: 8.53-46.27] more likely to deliver at the health facility compared to those whose husbands did not encourage them to do so.

Of all the perceived need-related variables only birth preparation [aOR: 1.75; 95% CI: 1.04-2.92] and the experience of complications during pregnancy or childbirth [aOR: 2.88; 95% CI: 1.67-4.98] had increased the odds of institutional delivery in the final model.

Among the perceived health service-related variables, only the availability of skilled health worker and the adequacy of physical facilities retained in the final model. Women believing that skilled health workers were always available were about 3 times [aOR: 2.70; 95% CI: 1.20-6.07] more likely to deliver at a health facility compared to those who perceived the health worker to be sometimes available, net of all other factors. Those who had no idea about the adequacy of the physical facilities of the health facility were far less likely to deliver there than those who perceived the health facility infrastructure to be inadequate [aOR: 0.14; 95% CI: 0.05-0.41].

Obviously, those who reported that it takes less than an hour to reach the nearest birthing facility by the available means of transportation were more than two times [aOR: 2.15; 95% CI: 1.26-3.69] as likely to deliver there compared to those reporting longer travel times.

## Discussion

### Significance of socio-economic and socio-demographic factors

Findings revealed that caste/ethnicity was the only socio-economic/demographic variable that statistically significantly influenced institutional delivery and retained in the final model. This is in contradiction to the findings of previous studies conducted in Nepal [31,32], where age, parity and education showed clear associations with institutional delivery. The reason may be that due to the way we categorized the castes/ethnicity variable and the distribution of castes/ethnicity in the study area, the differences between advantaged (i.e. Brahman/Chhetri, very few Gurung and Newar) and disadvantaged (i.e. disadvantaged Janjati- Chepangs/Tamang/Rai/Tharu, and Dalits) castes/ethnicity were much more pronounced compared to other studies. Another important reason may be that we included a specific decision-making variable (see next section), which is absent from most studies [31-34]. A study conducted in India concluded that economic status was more important for the decision on the place of delivery than even distance to a health facility, applicable particularly in private-for-profit health facilities [35]. At least in the setting of our study, this observation was not supported, which is likely to be a consequence of the introduction of free delivery services and maternity incentives in Nepal, as well as the expansion of birthing facilities to rural areas.

### Significance of the role of family in decision-making

Surprisingly, this study suggests that the likelihood of institutional delivery was greater when the decision on the place of delivery was taken jointly by the mother and other family members and even when other family members decided without the mother but together with the FCHV than when the mother decided by herself. It suggests that a woman’s autonomy alone is not a critical factor but interacts with others, such as the fact that many husbands in the study area are absent for work. Reaching a health facility can be difficult and, consequently, shy and young women may not wish to go to a health institution for delivery imposing this burden on their husbands’ family. Similar findings were observed in a study in Uganda [36], where the likelihood of institutional delivery was about 6 times greater when the decision was taken by both spouses together and about 5 times greater when it was taken together with other relatives than when the decision was taken by the women alone [37]. Similarly, evidence from Tanzania showed that the likelihood of institutional delivery was higher when both husband and wife valued the importance of institutional delivery and the significance of doctors compared to traditional birth attendants [38]. Our finding also echoes that of an overview article on maternal survival, which suggests that women are reluctant to use available services due to the difficulties in accessing transportation, people needed for company and finding it shameful to give birth in front of others [6].

Our finding that a husband’s explicit support for institutional delivery influences the chances of a child being born at a health facility adds further weight to the significant role of the family in decision-making. Similar findings regarding the importance of a husband’s support were obtained in studies from Tanzania [38], Bangladesh [39], and Uganda [40]. Overall, these results suggest that the husband and other family members do play a significant role in the decision on place of delivery and that campaigns to encourage institutional delivery should not only target women themselves but also their families.

### Significance of perceived need-related factors

Our study shows that being prepared for birth (with saving money being the most common practice and other recommended preparations being poorly applied) and the experience of complications during the current pregnancy or childbirth significantly increased the chances of institutional delivery. The importance of birth preparedness was previously reported for Nepal [41] and Bangladesh [42], and was well emphasized in low- and middle-income countries [43]. The issue of complications during pregnancy and childbirth merits particular attention, as according to the Nepal Maternal Mortality and Morbidity Study, 41% of maternal deaths in 2008/09 occurred in a health facility (an increase from 21% in 1998) compared to 40% taking place at home (a decrease from 67% in 1998) [44]. These numbers suggest that it is still primarily women with complications that are brought to a health facility and that many women seek care too late [45]. Indeed, among the maternal deaths that occurred in hospital in 2008/09, 83 percent were admitted as an emergency case in a critical condition [44].

Unlike studies in Nepal [31,32], and Ethiopia [46,47] the present study found no association of ANC use with the place of delivery. Likewise, ANC use did not emerge as a critical factor influencing institutional delivery in a study conducted in Uganda [36]. One possible explanation may be the fact that the experience of pregnancy complications increases the likelihood of a greater number of ANC check-ups, making the latter variable redundant in the final model. In summary, efforts to strengthen institutional delivery in Chitwan and other districts of Nepal should stress the importance of being prepared for birth and the advantages of an institutional delivery and independent of the immediate need for assistance due to complications during pregnancy and childbirth.

### Significance of perceived health service-related factors

A woman’s perception with respect to the constant availability of a skilled health worker and the adequacy of physical facilities was associated with a greater likelihood of institutional delivery. The international literature offers mixed results regarding the quality of health service and their influence on the place of delivery [48,49]. While the quality of health services always plays an important role, in a Nigerian study the competence of doctors/midwives and their 24-hour availability to provide maternity service was critical [48], while a Bangladeshi study indicated that more value was attached to the respectful behaviour of health workers rather than their technical skills [49].

In many rural communities in Chitwan the birthing facilities were only built during the last two years and continue to be characterised by very poor infrastructure and by few of their staff, mostly auxiliary nurse midwives, having received the training to become a skilled birth attendant. This may explain why women valued the quality of personnel and infrastructure more than the behaviour of health workers. Ultimately, all aspects of quality of healthcare including an adequate facility characterised by adequate rooms for accommodation, service delivery, water, light; the availability of drugs and other commodities and the provision of services at all hours by competent and respectful healthcare workers will be critical in increasing institutional delivery rates.

### Significance of distance to birthing facility

In the current study women living within one hour’s distance from a birthing facility were more likely to give birth there than women living further away. This finding is consistent with another study conducted in Nepal [32], as well as studies undertaken in Tanzania [50] and Bangladesh [51]. Therefore, despite the government’s efforts to establish facilities nearby and to provide incentives, accessibility continues to be a problem and requires further attention in programmes to promote institutional delivery.

### Strengths and limitations

This study is not free from some methodological limitations but equally shows some important strength. Like all cross-sectional studies, it identifies associations of different factors with institutional delivery but cannot establish causal relationships.

The study area was confined to six rural VDCs of Chitwan district, characterized by relatively low institutional delivery rates. While this may limit the generalizability of findings to other settings in Chitwan district and the country as a whole, nearly three quarters of the study population were of disadvantaged ethnicity, which is the priority group targeted by the government of Nepal in the provision of safe motherhood services [52]. Importantly, we ensured that all eligible mothers that had given birth during a pre-defined period were identified and recruited into the study by consulting FCHVs, reviewing their records and making additional enquiries with other local residents. The non-response rate of eligible mothers who could not be recruited as they had gone to their maternal home, to work or could not be accessed due to heavy rains did not exceed the 10 percent included in the sample size estimation. It is unlikely that any bias was introduced through non-responders, as they were of similar socio-economic status and from the same ethnic groups as the included mothers.

## Conclusions

This study, conducted in rural Chitwan district, shows that socio-demographic, socio-cultural, and health service-related factors interact in determining the uptake of birthing facilities by pregnant women and their families. One finding worth highlighting is the critical role that husband’s support plays in the decision for institutional delivery. Gender roles limiting women’s involvement in decision-making, young women having no access to material resources and higher illiteracy rates make women dependent on their husbands and other family members in having access to maternal health services, including delivery at a health facility. In addition, in some rural areas without an established transportation infrastructure, women have to be carried by their husbands to reach health facilities.

Therefore, in the geographical and socio-cultural context of Nepal, programmes to promote institutional delivery must, on the one hand, work through the empowerment of women and, on the other hand, specifically target husbands (and, to a lesser extent, other family members) and strengthen their involvement. Other important implications for policy and practice emerging from this study are a need to focus on families from disadvantaged castes, raising awareness of complications during pregnancy and childbirth while promoting childbirth in health facilities independent of the occurrence of such complications and, critically, ensuring physical accessibility to the birthing facilities. In relation to strengthening health services themselves, physical facilities must urgently be improved to attract women to give birth and the skills of health workers, in particular in relation to skilled birth attendant training, must be improved.

This cross-sectional study has investigated the effects of a broad range of factors on the place of delivery and has suggested some explanations on how these different factors exert their influence. It does, however, not offer in-depth insights into the pathways affecting decision-making and how these could be addressed through public health programmes in the most effective way. Therefore, a qualitative study that explores in more detail how these different factors, in particular those relating to decision-making by the couple and the wider family is merited. Moreover, the effectiveness of many of the different programmes to reduce maternal deaths, including the provision of birthing centres, free maternity services and financial incentives currently in place in Nepal, has not been evaluated to date. Their successful implementation and further improvement would benefit from high-quality operational research, in particular using randomised as well as natural experiment studies. Much remains to be done to optimise maternal health services in an effort to reduce unnecessary maternal deaths in Nepal and the world.
